# Hyperprogressive Disease In a Metastatic Renal Cell Carcinoma Patient After Receiving Immune Checkpoint Inhibitors: A Case Report

**DOI:** 10.7759/cureus.30194

**Published:** 2022-10-11

**Authors:** Mohammad Alkader, Rashed Altaha, Lean Alkhatib, Eslam H Jabali, Mohammad S Alsoreeky

**Affiliations:** 1 Department of Clinical Oncology, Jordanian Royal Medical Services, Amman, JOR; 2 Department of Internal Medicine, Jordanian Royal Medical Services, Amman, JOR; 3 Department of Diagnostic Radiology, Jordanian Royal Medical Services, Amman, JOR

**Keywords:** ctla-4 inhibitors, pdl-1 inhibitor, programmed death-1 (pd-1) inhibitors, immune escape, hyperprogression after immunotherapy, metastatic renal cell carcinoma with sarcomatoid features, hyperprogressive disease, immune checkpoint inhibitors (icis), ipilimumab nivolumab

## Abstract

Owing to their survival benefits, immune checkpoint inhibitors (ICIs) have emerged as the mainstay treatment for several types of malignant tumors including renal cell carcinoma (RCC). However, the usage of ICIs such as nivolumab, ipilimumab, and atezolizumab can be complicated by unexpected rapid clinical deterioration and acceleration of tumor growth. This adverse event is called hyperprogressive disease (HPD) with an incidence rate of 10-20%. Since its first description in 2016, efforts have been made to identify and predict this phenomenon.

We report a case of a 34-year-old female patient diagnosed with metastatic renal cell carcinoma (mRCC) with sarcomatoid features. She underwent a left radical nephrectomy followed by combination ICIs (nivolumab/ipilimumab) therapy. However, she presented with a rapid clinical deterioration shortly after receiving her second cycle of ICIs. The radiological assessment showed new multiple bilateral lung nodules, new multiple mediastinal and left hilar lymph node involvement, and two focal areas of new appearance involving the left aspect of L3 lumbar vertebrae and left ischial bone. The diagnosis of HPD was made. Unfortunately, the patient died soon following her second infusion of the nivolumab/ipilimumab combination.

## Introduction

ICIs are one of the major developments in cancer therapy. They act by blocking the interactions between cancer cells and the immune system, which enables the immune system, particularly T lymphocytes, to eliminate cancer cells more effectively. To date, seven ICIs are approved to manage different types of cancers such as RCC, melanoma, non-small cell lung cancer (NSCLC), and head and neck squamous cell carcinoma (HNSCC) [1]. Significant long-lasting responses were observed among a subset of cancer patients receiving ICIs. However, apart from tumor resistance, ICIs can be complicated by hyperprogressive disease (HPD) [2]. 

HPD is defined as a rapid clinical deterioration of the disease following immunotherapy. It occurs in around 10-20% of cases [3]. An important distinction is to differentiate HPD from another distinct novel response to ICIs called pseudoprogression (PSP). PSP is a transient form of progression with an apparent initial increase in tumor size or appearance of new lesions, followed by a decrease in tumor burden and shrinkage. It indicates treatment efficacy, likely from inflammatory cell infiltration instead of tumor cell proliferation, and does not require the discontinuation of ICI treatment. In contrast, HPD can be defined as an accelerated tumor growth rate, with an increase in the absolute mass of tumor cells in a relatively short time. It depicts ineffective treatment with ICIs, resulting in rapid clinical deterioration and poor survival outcomes, and requires prompt discontinuation of ICIs treatment [4]. A new model called nomogram was established to predict the occurrence of HPD in lung cancer patients. This model calculates a score based on lactate dehydrogenase (LDH), erythrocyte sedimentation rate (ESR), and mean corpuscular hemoglobin concentration (MCHC) [3]. The mechanism of HPD includes both alterations at the level of tumor cells and the tumor microenvironment. However, there are no current definitive diagnostic criteria for HPD. Patients who develop HPD have significantly lower overall survival outcomes in comparison with non-HPD patients [5].

The sixth-most prevalent cancer in males and the ninth-most prevalent in females is renal cell carcinoma (RCC). The standard management of early-stage disease is partial or radical nephrectomy. High-risk patients can be treated with sunitinib as adjuvant therapy. However, the administration of sunitinib is limited by its toxicity. Anti-vascular endothelial growth factor (VEGF) agents and ICIs have been developed over the last two decades and have shown better outcomes than sunitinib in patients with advanced renal cell carcinoma (aRCC). Currently, many clinical trials are studying the role of ICIs as both adjuvant and neoadjuvant therapies [6].

Nivolumab is an immunoglobulin (IgG4) programmed death-1 (PD-1) checkpoint inhibitor approved by the Food and Drug Administration (FDA) for managing aRCC in patients who received anti-angiogenic therapy. Nivolumab was found to be well tolerated. However, it can be complicated by generalized weakness, decreased appetite, nausea, diarrhea, itching, and rash. The overall response rate (ORR) was found to be better in intermediate to poor risk aRCC patients receiving nivolumab plus ipilimumab combination when compared to those receiving sunitinib [7]. Here, we present a fatal case of HPD in a patient with aRCC who received a combination of ICIs (nivolumab/ipilimumab).

## Case presentation

**Figure 1:**
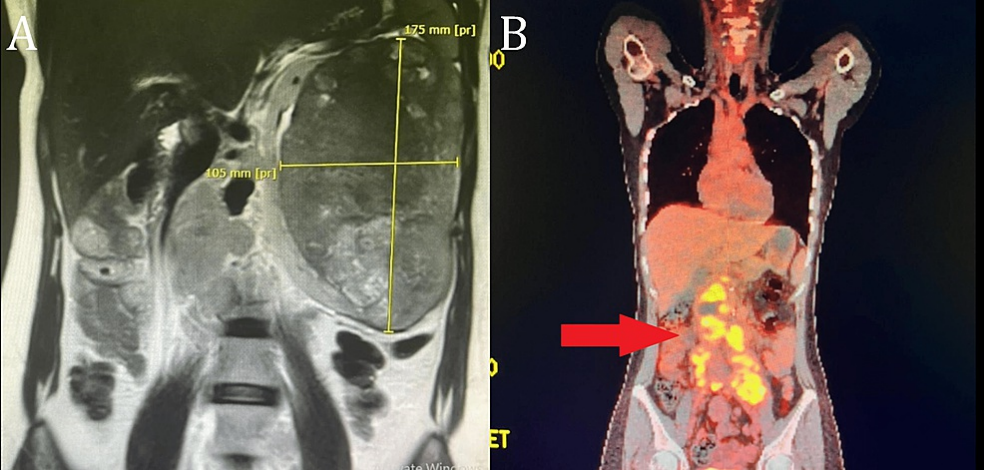
(A) Renal MRI Vefore Left Radical Nephrectomy; (B) Baseline PET/CT Scan Before ICI Treatment. (A) Left heterogeneous enhancing renal mass measuring (175x105 mm). (B) Numerous retroperitoneal lymph nodes involving left para-aortic, aortocaval, and retrocaval lymph nodes. ICI: immune checkpoint inhibitors

A 34-year-old female patient was diagnosed with mRCC with sarcomatoid features. She presented to our department complaining of hematuria associated with a palpable left flank mass and with no identifiable risk factors such as smoking history, obesity, genetic and hereditary factors, or exposure to chemicals known to cause RCC. Her baseline laboratory investigations before ICIs treatment showed normocytic anemia and elevated erythrocyte sedimentation rate (ESR) (Table 1). Based on the Memorial Sloan-Kettering Cancer Center (MSKCC) model and International Metastatic RCC Database Consortium (IMDC) prognostic criteria, she was considered to be an intermediate-risk patient with an intermediate prognosis. Her Eastern Cooperative Oncology Group (ECOG) performance and Karnofsky performance status (KPS) were 0 and 100% respectively. Renal magnetic resonance imaging (MRI) showed left heterogeneous enhancing renal mass measuring (175x105 mm) (Figure 1A). Her baseline positron emission tomography/computed tomography (PET/CT) scan (Figure 1B) showed numerous retroperitoneal lymph nodes involving left para-aortic, aortocaval, and retrocaval lymph nodes with the largest measuring 31x30 mm. Also, her PET/CT scan showed hypermetabolic mesenteric and left retroperitoneal deposits, some of them attached to the left psoas muscle.

**Table 1:** Baseline Laboratory Investigations Before ICIs Treatment ICI: immune checkpoint inhibitors; WBC: white cell count; Hb: hemoglobin; MCV: mean corpuscular volume; MCHC: mean corpuscular hemoglobin concentration; PLT: platelet count; LDH: lactate dehydrogenase

Test	Result	Units	Reference Range
Wbc	8.0	μL	4-11
Neutrophils	5.4	μL	2-7
Hb	10.5	g/dL	10.5
MCV	88.7	fL	88.7
MCHC	32.9	g/dL	32-36
PLT	424	μL	150-450
LDH	200	U/L	14-240
ESR	55	mm/hr	0-20
Calcium	9.61	mg/dL	8.6-10.5

The patient underwent a left radical nephrectomy. Clear cell renal carcinoma with sarcomatoid features was confirmed by the histologic examination. Following her surgery, the patient was offered to receive first-line combination therapy with ICIs (nivolumab 3 mg/kg IV over 30 minutes, followed by ipilimumab 1 mg/Kg IV over 30 minutes, repeat cycle every three weeks for four cycles). Around 14 days after her second cycle of ICIs (i.e. five weeks from the beginning of ICIs), she presented to the emergency department with a significant clinical deterioration and an ECOG performance of 2. 

**Figure 2:**
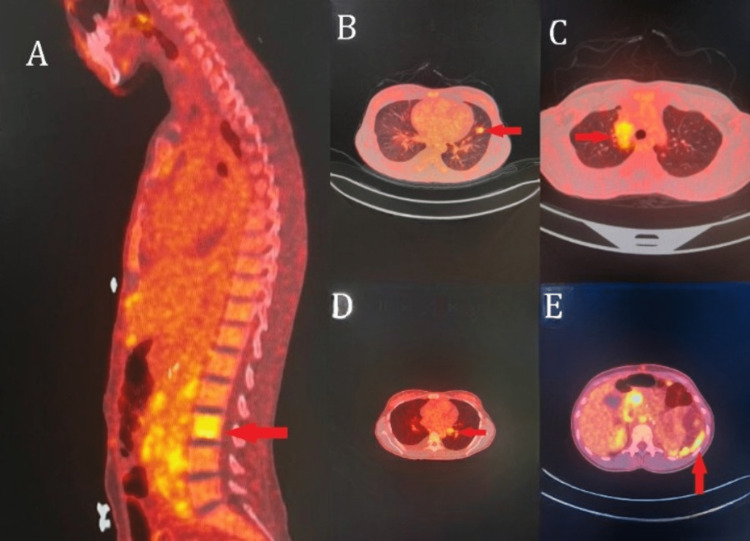
PET/CT Scan Five Weeks After ICI Treatment. (A) Evidence of a new area with abnormally increased FDG uptake involving the left aspect of L3 lumbar vertebrae. (B) New multiple bilateral lung nodules, variable in size, showing a variable degree of abnormal increased FDG uptake being more prominent in the upper lobe of left lung SUV max 3.4. (C) New mediastinal lymph node involvement showing abnormal increased FDG uptake. (D). New left hilar lymph node showing abnormal increased FDG uptake. (E) Evidence of new multiple peritoneal soft tissue densities showing abnormal increased FDG uptake. ICI: immune checkpoint inhibitor; FDG: fludeoxyglucose; SUV: standardized uptake value

Her follow-up PET/CT scan showed the following: (1) new multiple bilateral lung nodules that are variable in size and demonstrate a variable degree of abnormally increased fludeoxyglucose (FDG) uptake, more prominent in the upper lobe of the left lung with standardized uptake value (SUV) max 3.4; (2) new multiple mediastinal and left hilar lymph nodes involvement showing an abnormal increased FDG uptake as well; (3) evidence of new multiple peritoneal soft tissue densities showing an abnormal increased FDG uptake; (4) two focal areas of new appearance with an abnormal increased FDG uptake involving the left aspect of L3 lumbar vertebrae and left ischial bone corresponding to CT scan findings with sclerotic bony lesions (Figure 2).

**Figure 3:**
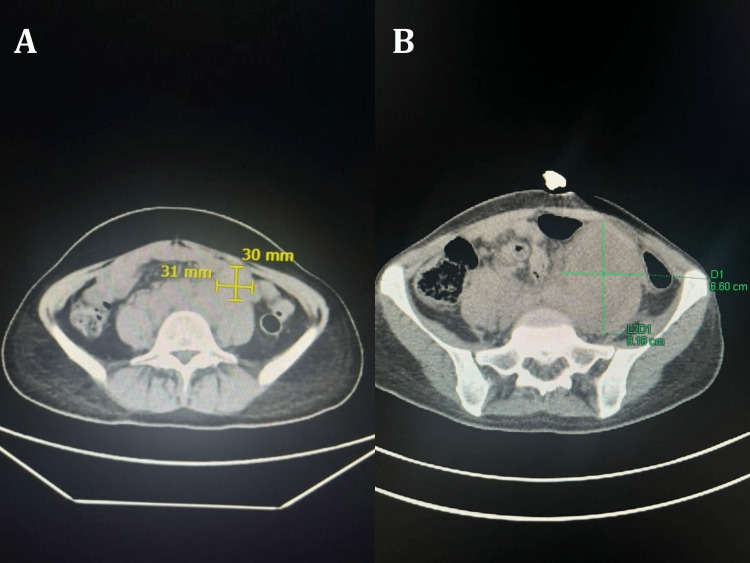
> 258% Increase In The Total Diameter Of The Left Common Iliac Lymph Node. (A) Left common iliac lymph node measuring (31x30 mm) before ICI treatment. (B) > 258% increase in the total diameter of the common iliac lymph node after ICI treatment. ICI: immune checkpoint inhibitor

The diagnosis of HPD was made based on the suggested criteria of Lo Russo; she met four of the five criteria: (1) time to treatment failure of five weeks; (2) restaging imaging was done 1.5 months after starting the combination ICIs and showed a 258% increase in the size of the left common iliac lymph node from pre-immunotherapy imaging (Figure 3); (3) distribution to the lung, mediastinal lymph nodes, left hilar lymph nodes, and bone; (4) clinical deterioration from ECOG performance status of 0 to 2. Since the patient underwent a left radical nephrectomy, the affected organ, she scored 4 out of 5. ICIs combination therapy was discontinued and shifted to cabozantinib 60 mg daily for three months. Unfortunately, the patient died soon after her second infusion of the nivolumab/ipilimumab combination.

## Discussion

One of the hallmarks of cancer cells is immune escape [8]. The immune system, specifically T lymphocytes, plays a significant role in fighting cancer cells. Immunosurveillance is a term that describes the immune system while recognizing and destroying cancerous cells. Cancer cells can evade immune destruction from tumor-reactive T lymphocytes. So efforts have been made to understand T cell immunobiology, and they have been crucial in developing therapeutic strategies to overcome immune escape. In particular, the development of immune checkpoint inhibitors has revolutionized the field of cancer therapeutics, designed to interfere with inhibitory pathways that naturally restrain T cell reactivity. Immune checkpoint blockade has proven to be an effective strategy for enhancing tumor-reactive T cells. Checkpoint inhibitors acting against cytotoxic T-lymphocyte-associated antigen-4 (CTLA-4), PD-1, and programmed death-ligand 1 (PD-L1) have shown promising and outstanding responses in many cancer patients in recent years [9]. To date, there are seven FDA-approved ICIs classified into three different categories: PD-1 inhibitors, such as pembrolizumab, nivolumab, and cemiplimab; PDL-1 inhibitors such as durvalumab, atezolizumab, and avelumab; and CTLA-4 inhibitors such as ipilimumab [10]. Similar to autoimmune diseases, ICIs might cause immune-related adverse events (irAEs). Any organ system can be involved, and these reactions can range from mild to moderate. However, life-threatening events can occur. Steroids are the mainstay of irAEs management. However, alternative immunosuppressants can be considered in steroid-resistant cases [11].

The wide application of ICIs has led to the observation of a recent pattern of poor response where the patient rapidly experiences clinical deterioration associated with poor survival outcomes. It was called HPD and was described for the first time in a wall newspaper in 2016. Since then, more interest grew in the scientific community to establish a common ground for physicians to identify and diagnose HPD [5]. Several hypotheses are currently under investigation to find a plausible underlying mechanism of HPD; murine double minute 2/4 (MDM2/MDM4) amplification, epidermal growth factor receptor (EGFR) mutations, T cells, B cells, fragment crystallizable (Fc) receptor, and cluster of differentiation 4 (CD4+) regulatory T cells (Treg) are involved in the pathogenesis of HPD [12]. The management of HPD begins with the discontinuation of ICIs. In theory, HPD signifies rapid tumor proliferation at the cell cycle level. Therefore, one could consider the possibility of administering cell cycle-specific chemotherapeutic agents, such as taxanes (docetaxel, paclitaxel) and vinca alkaloids (vincristine and vinblastine) [12].

Lo Russo G criteria are one of the suggested diagnostic models for HPD. It requires three out of five of the following: (1) TTF (time to failure of treatment) of less than two months; (2) an increase of 50% in the total diameter of the target lesions; (3) at least two new lesions develop in an affected organ; (4) distribution to a new organ; and (5) clinical deterioration to performance status (PS) ≥ 2 [13]. Matos et al.'s other diagnostic criteria for HPD require the development of HPD in the first eight weeks of starting ICIs with an increase of 40% in the total diameters of the target lesions or the appearance of new lesions in at least two distinct organs [14]. To put things into perspective, we considered what would the growth rates look like for untreated primary and metastatic lesions of RCC. According to a study done by Oda et al., the growth rate for primary lesions ranged from 0.10 to 1.35 centimeters (cm)/year and from 0.08 to 7.87 cm/year for metastatic lesions. RCC is a slow-growing tumor and the range variation of growth in cm is related to histologic grade and histologic subtype. Notice the time unit used in the study is per year, whereas HPD is defined by an accelerated tumor growth rate that occurs in a much shorter time (i.e. within two months of ICIs treatment) [15]. Given these challenges, the HPD continues to be an intuition of the treating physician rather than a phenomenon that is defined in a standardized way [16].

RCC with clear cell morphology is the most common type of RCC. The Memorial Sloan Kettering Cancer Center (MSKCC) and the International Metastatic RCC Database Consortium (IMDC) models are the most common prognostic systems in stratifying the risk of metastatic ccRCC. The MSKCC model includes serum LDH, hemoglobin, Karnofsky performance status, corrected serum calcium level, and time from diagnosis to management. The IMDC system is similar to the MSKCC model. However, it uses neutrophils and platelets count instead of LDH level [6]. 

The mainstay treatment for early-stage disease is partial or radical nephrectomy. For the treatment of those who are at high risk, sunitinib can be given as adjuvant therapy. However, sunitinib's toxicity limits its administration. RCC is a suitable candidate for ICIs because it is one of the cancers with a significant immune infiltrate. Over the past 20 years, anti-VEGF medications and ICIs have improved outcomes in patients with advanced renal cell carcinoma (aRCC) [6].

## Conclusions

HPD is still an evolving concept. To date, oncologists depend on their intuition combined with radiological and clinical assessments of their treating individuals. We hope our case will help future oncologists to create internationally acceptable evidence-based guidelines that can define and predict HPD following ICI treatment. By that time, hopefully, this will translate to developing effective methods to prevent HPD and its associated devastating outcomes in a subset of patients who are at risk of HPD.

## References

[1] Okan Cakir M, Kirca O, Gunduz S, Ozdogan M (2019). Hyperprogression after immunotherapy: A comprehensive review. J BUON.

[2] Adashek JJ, Kato S, Ferrara R, Lo Russo G, Kurzrock R (2020). Hyperprogression and Immune Checkpoint Inhibitors: Hype or Progress?. Oncologist.

[3] Cao S, Zhang Y, Zhou Y (2022). A nomogram for predicting hyperprogressive disease after immune checkpoint inhibitor treatment in lung cancer. Transl Lung Cancer Res.

[4] Patel K, Mukhi H, Patel A (2022). Differentiating pseudoprogression from hyperprogression in patients treated with immunotherapies. Targeted Therapies in Oncology.

[5] Ding P, Wen L, Tong F, Zhang R, Huang Y, Dong X (2022). Mechanism underlying the immune checkpoint inhibitor-induced hyper-progressive state of cancer. Cancer Drug Resist.

[6] Yu EM, Linville L, Rosenthal M, Aragon-Ching JB (2021). A contemporary review of immune checkpoint inhibitors in advanced clear cell renal cell carcinoma. Vaccines (Basel).

[7] Ochoa CE, Joseph RW (2018). Nivolumab in renal cell carcinoma: current trends and future perspectives. J Kidney Cancer VHL.

[8] Hanahan D (2022). Hallmarks of cancer: new dimensions. Cancer Discov.

[9] Hargadon KM, Johnson CE, Williams CJ (2018). Immune checkpoint blockade therapy for cancer: An overview of FDA-approved immune checkpoint inhibitors. Int Immunopharmacol.

[10] Shiravand Y, Khodadadi F, Kashani SM (2022). Immune checkpoint inhibitors in cancer therapy. Curr Oncol.

[11] Morgado M, Plácido A, Morgado S, Roque F (2020). Management of the adverse effects of immune checkpoint inhibitors. Vaccines (Basel).

[12] Liu X, Qiao L (2022). Hyperprogressive disease in malignant carcinoma with immune checkpoint inhibitor use: a review. Front Nutr.

[13] Toki MI, Syrigos N, Syrigos K (2021). Hyperprogressive disease: A distinct pattern of progression to immune checkpoint inhibitors. Int J Cancer.

[14] Brambilla M, Russo GL, Ferrara R, Manglaviti S, Garassino MC, Occhipinti M (2020). Is hyperprogressive disease a specific phenomenom of immunotherapy?. Explor Target Antitumor Ther.

[15] Oda T, Miyao N, Takahashi A (2001). Growth rates of primary and metastatic lesions of renal cell carcinoma. Int J Urol.

[16] Matos I, Martin-Liberal J, García-Ruiz A (2020). Capturing hyperprogressive disease with immune-checkpoint inhibitors using RECIST 1.1 criteria. Clin Cancer Res.

